# Patterns of Comorbidity and Multimorbidity Among Patients With Multiple Sclerosis in a Large US Commercially Insured and Medicare Advantage Population

**DOI:** 10.36469/001c.38669

**Published:** 2022-11-21

**Authors:** Dingwei Dai, Ajay Sharma, Amy L. Phillips, Carroline Lobo

**Affiliations:** 1 CVS Health Clinical Trial Services LLC, Woonsocket, Rhode Island, USA; 2 Health Economics & Outcomes Research, EMD Serono, Rockland, Massachusetts, USA

**Keywords:** multiple sclerosis, comorbidity, multimorbidity, managed care, disease management, decision-making, retrospective observational study

## Abstract

**Background:** Comorbidities are common in patients with multiple sclerosis (MS), thus increasing the complexity of disease management and economic burden and worsening their prognosis and quality of life. Real-world evidence comparing comorbidities and multimorbidity patterns of commercially insured vs Medicare enrollees with MS is lacking. **Objective:** To evaluate the patterns of comorbidity and multimorbidity among patients with MS in a US commercially insured and Medicare Advantage population. **Methods:** This retrospective observational cohort study was conducted using Aetna health claims data from January 1, 2015, to October 31, 2019. Eligibility criteria were (1) at least 3 MS-related inpatient/outpatient (ICD-10-CM: G35), or disease-modifying therapy claims within 1 year (date of first claim = index date); (2) Aetna commercial health plan or Medicare Advantage medical and pharmacy benefits at least 12 months pre-/post-index; and (3) age 18 and older. Commercially insured patients, Medicare Advantage patients younger than 65 years of age, and Medicare Advantage patients 65 years and older were compared. **Results:** Among 5000 patients (mean [SD] age, 52.6 [12.9]; 75.2% female), 53% had commercial insurance and 47% had Medicare Advantage (59.2% disabled age <65). Medicare Advantage patients were older (age <65: 53.3 [7.9]; age ≥65: 70.8 [5.2]) vs commercial (age, 45.7 [10.2]), had greater comorbidity burden (Charlson Comorbidity Index; age <65: 1.17 [1.64], age ≥65: 1.65 [1.95]) vs commercial (0.53 [1.02]) (all *P* < .0001). Symptoms specific to MS (ie, malaise, fatigue, depression, spasms, fibromyalgia, convulsions) were more common among patients younger than 65 (all *P* < .0001). Age-related and other comorbidities (ie, hypertension, hyperlipidemia, dyspepsia, osteoarthritis, osteoporosis, glaucoma, diabetes, cerebrovascular, cancer) were more common among patients 65 years and older Medicare Advantage (all *P* < .0001). Multiple comorbidities were highly prevalent (median, 4 comorbidities), particularly among Medicare Advantage patients younger than 65 (median, 6) and Medicare Advantage patients 65 and older (median, 7). **Conclusions:** Comorbidities and multimorbidity patterns differed between patients with MS with commercial insurance and patients with Medicare Advantage. Multimorbidity was highly prevalent among patients with MS and should be considered in the context of clinical decision making to ensure comprehensive MS management and improve outcomes.

## INTRODUCTION

Evidence has shown that comorbidities among patients with multiple sclerosis (MS) may delay diagnosis^1^; increase severity and disability progression,^1,2^ relapse,^3^ hospitalization,^4^ death,^5^ and healthcare costs^6^; and reduce health-related quality of life.^7^ There is considerable variability across comorbidity studies regarding study populations and methods.^8^ A systematic review of 249 articles found the most prevalent comorbidities in MS were depression (23.7%), anxiety (21.9%), hypertension (18.6%), hyperlipidemia (10.9%), and lung disease (10.0%).^8^ Recent studies evaluating comorbidity have evaluated large populations to provide more representative and valid estimates.^9^ Analyses of mortality and comorbidities in MS (n = 15 684) and non-MS (n = 78 420) patients from the US Department of Defense database found that sepsis (event rate ratio: 5.7), ischemic stroke (3.8), attempted suicide (2.4), ulcerative colitis (2.0), lymphoproliferative disorders (2.2), and melanoma (1.7) were more common with MS.^10^ A 2006-2014 analysis of 5 million commercially insured US patients found the most common comorbidities were hyperlipidemia and hypertension (range, 25.9%-29.7%), gastrointestinal disease (range, 18.4%-21.2%), and thyroid disease (range, 12.9%-17.1%).^11^ A propensity score-matched study using Medical Expenditure Panel Survey (2005-2015) data found that significantly greater proportions of patients with MS had arthritis (42.1% vs 30.1%), depression (29.7% vs 13.3%), anxiety (17.1% vs 8.5%), osteoporosis (5.7% vs 2.4%), and anemia (5.5% vs 1.8%), compared with controls (all *P* < .05).^12^

Approximately 25% to 30% of US patients with MS are Medicare beneficiaries; however, limited information exists on this population.^13^ Previous studies have documented the high costs borne by traditional Medicare patients with MS, and large proportions of the costs were associated with comorbidity.^13^ There is a growing need by researchers and policy makers to understand the role of Medicare Advantage.^14^ Enrollment in Medicare Advantage is growing rapidly, increasing from 26% of Medicare in 2012 to 42% in 2021, now including more than 24 million beneficiaries.^14,15^ Published evidence comparing patient characteristics, comorbidities, and multimorbidity patterns of commercially insured vs Medicare Advantage enrollees with MS is lacking. Such knowledge may be important in optimizing disease management and clinical outcomes and minimizing healthcare costs. This study examined patient characteristics, comorbidities, and multimorbidity patterns in commercially insured and Medicare Advantage patients with MS using a large US administrative claims database.

## METHODS

### Study Design and Data Source

This retrospective observational cohort study evaluated adult patients with MS using Aetna’s administrative claims data from January 1, 2015, to October 31, 2019 (**Supplementary Figure S1**). The database includes more than 20 million members and contains patient enrollment and inpatient and outpatient medical and pharmacy claims data for fully insured commercial health plan and Medicare Advantage members.^16^ As this nonexperimental study did not require direct patient identification, a Limited Data Set, as defined by the Health Insurance Portability and Accountability Act (HIPAA) Privacy Rule, was used. The study was approved by an institutional review board.

### Patient Selection

Adult patients with MS were identified between January 1, 2016, and October 31, 2018 (index period), using a validated algorithm that required at least 3 MS-related hospitalizations (*International Classification of Diseases, Tenth Revision, Clinical Modification* [ICD-10-CM]: G35), outpatient visits (ICD-10-CM: G35), or prescriptions for an MS disease-modifying therapy (DMT) within 1 year.^17^ DMTs approved by the US Food and Drug Administration by 2019 were considered, including interferon beta, glatiramer acetate, fingolimod, mitoxantrone, cladribine tablets, daclizumab, dimethyl fumarate, siponimod, teriflunomide, alemtuzumab, ocrelizumab, and natalizumab. As natalizumab is also approved for inflammatory bowel disease (IBD), claims for natalizumab where the individual also had ICD-10-CM codes for IBD were not included, to prevent potential misclassification.^17^

The index date for each patient was defined as the earliest service/claim date with evidence of MS (MS diagnosis or DMT). Patients with MS were eligible if they had an Aetna fully insured commercial health plan or Medicare Advantage with medical and pharmacy health insurance benefits for at least 12 months pre-index (baseline period) and 12 months post-index (follow-up period). Patients in Aetna’s Compassionate Care Program or hospice care were excluded as they did not necessarily have as thorough documentation of comorbidity.

### Patient Baseline Demographic and Clinical Characteristics

Patient demographic characteristics evaluated included age at index, sex, US geographic region (Midwest, Northeast, South, and West), rural or urban residence, and median household income. Household income was estimated by merging 2010 US Census data to the claims using zip codes. Incident cases were defined as patients with MS without any evidence of MS (ICD-10-CM: G35, DMT National Drug Code) during baseline. DMT treatment was identified as any pharmacy claims for DMT during follow-up.

### Assessment of MS-Related Conditions and Symptoms

Multiple sclerosis–related conditions and symptoms identified included abnormality of gait; ataxia, burning/numbness/tingling sensations, convulsions, depression, and fecal incontinence; fibromyalgia/myalgia and myositis, malaise and fatigue, optic neuritis, spasms, trigeminal neuralgia, urinary incontinence, and voice disturbances. These conditions and symptoms were selected based on the systematic review of published medical literature^18^ and were defined using ICD-10-CM codes at any positions in the claims.

### Assessment of Comorbidity and Multimorbidity

In patients with MS, comorbidity refers to 1 or more chronic conditions that occur together with MS. Presence of comorbidity was assessed using all available data (any positions in inpatient and outpatient claims) prior to and including the index date (**Online Supplementary Material, Figure S1**). Comorbidities evaluated were those most common among patients with MS based on published literature^8,11,18–22^ and the Aetna patient profile database.^16,23^ The comorbid conditions were defined using previously described methods using ICD-10-CM diagnosis codes (**Online Supplementary Material, Table S1**).^23,24^ The Charlson Comorbidity Index (CCI),^25^ a validated approach summarizing disease burden and predicting mortality risk and high healthcare costs,^26,27^ was also calculated. Multimorbidity was defined as the presence of at least 2 comorbid conditions within a patient.^28,29^ The number of comorbid conditions was a count of all comorbid conditions in each patient.

### Statistical Analyses

Demographics, clinical characteristics, and comorbidities were analyzed descriptively. Means (SD) or medians (interquartile range) were reported for continuous variables, and frequencies (%) were reported for categorical variables. Comparisons were made among commercially insured patients, Medicare Advantage patients younger than 65 years, and Medicare Advantage patients 65 years and older. Kruskal-Wallis tests for continuous variables and χ^2^ tests for categorical variables compared differences among payer types. Data management and statistical analyses were conducted using SAS version 9.4 (SAS Institute Inc, Cary, North Carolina). *P* values were 2-sided, with *P* < .05 considered statistically significant; however, multiplicity was not addressed, so all *P* values are descriptive.

## RESULTS

### Patient Baseline Demographic Characteristics

**Figure 1.**
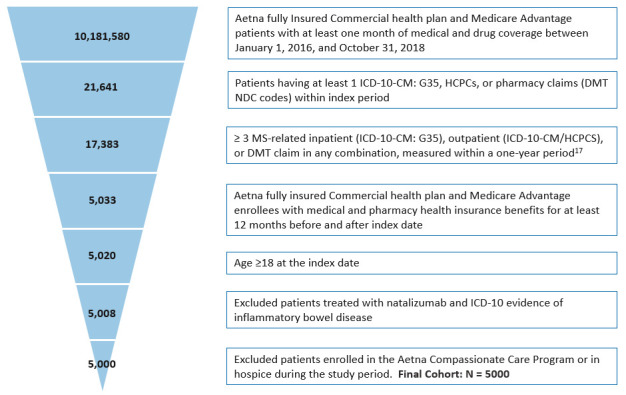
Patient Selection from Aetna Healthcare Claims Databases Abbreviations: DMT, disease-modifying therapy; HCPCS, Healthcare Common Procedure Coding System; ICD-10-CM, *International Classification of Diseases, Tenth Revision, Clinical Modification*; MS, multiple sclerosis; NDC, National Drug Code; SSDI, Social Security Disability Insurance.

A total of 5000 patients met eligibility criteria (**Figure 1**). Patient demographics, overall and by payer type, are presented in **Table 1**. Among 5000 patients, median age was 53 years, 75.2% were female, 34.3% were from the Northeast United States, 39.8% lived in rural areas, and median household income was $62 566. Only 20.8% of patients were incident cases (ie, no evidence of MS diagnosis or DMT in the baseline); the remaining 79.2% were prevalent MS cases. Only 55.3% of patients had DMT prescription claims during the 1-year follow-up. Commercially insured patients constituted 53.3% of the study population; the remaining 46.7% were Medicare Advantage patients. Among Medicare Advantage patients, 59.2% were disabled, younger than 65 years, and eligible for Medicare Advantage through the Social Security Disability Insurance; the remaining 40.8% were elderly beneficiaries eligible for Medicare Advantage at age 65 years and older.

**Table 1.** Patient Baseline Demographic and Clinical Characteristics of Patients with MS by Payer Type^a^

**Characteristics**	**Overall** **(N = 5000)**		**Payer Type**			
		**Commercial (n = 2667)**		**Medicare Advantage**		***P* Value^a^**
			**Overall** **(n = 2333)**	**Age <65 y** **(n = 1382)**	**Age ≥65 y (n = 951)**	
Age						
Mean (SD)	52.60 (12.90)	45.73 (10.20)	60.45 (11.07)	53.33 (7.99)	70.81 (5.16)	NA^b^
Median (IQR)	53 (44-61)	47 (39-54)	62 (53-68)	55 (48-60)	69 (67-74)	
Sex, %						.3353
Male	24.8	25.2	24.4	25.3	22.9	
Female	75.2	74.8	75.6	74.7	77.1	
Geographic region, %						<.0001
Midwest	22.3	12.7	33.3	32.1	35.1	
Northeast	34.3	33.2	35.6	34.6	37.1	
South	32.0	38.3	24.8	27.3	21.0	
West	11.4	15.8	6.3	6.0	6.8	
Urban-rural, %						<.0001
Urban	31.1	37.8	23.5	25.3	20.8	
Suburban	29.1	27.2	31.3	31.2	31.4	
Rural	39.8	35.1	45.2	43.5	47.7	
Median household income, $^c^						<.0001
Mean (SD)	66 866 (24 762)	72 054 (26 329)	60 969 (21 376)	59 872 (20 929)	62 561 (21 923)	
Median (IQR)	62 566 (48 491-80 526)	68 387 (52 752-87 467)	56 723 (45 772-72 505)	56 058 (45 016-71 061)	58 715 (47 230-74 473)	
CCI						<.0001
Mean (SD)	0.92 (1.49)	0.53 (1.02)	1.37 (1.79)	1.17 (1.64)	1.65 (1.95)	
Median (IQR)	0 (0-1)	0 (0-1)	1 (0-2)	1 (0-2)	1 (0-2)	
MS case, %						<.0001
Incidence	20.8	25.2	15.7	11.5	21.8	
Prevalence	79.2	74.8	84.3	88.5	78.2	
DMT, %	55.3	67.3	41.5	51.1	27.7	<.0001

Means, SD, and medians (IQRs) were reported for continuous variables. Frequencies (percentages) were reported for categorical variables.

^a^Comparison among commercial, Medicare Advantage age <65 years, and Medicare Advantage age ≥65 years. Statistical significance was assessed with Kruskal-Wallis test for continuous variables and χ2 test for categorical variables. The *P* values presented in this table have not been corrected for multiplicity and should be considered descriptive in nature.

^b^Comparisons were not possible as plan eligibility was based on age.

^c^The estimated median household income represents the patient’s neighborhood median household income obtained from US Census data.

Patients with Medicare Advantage were older (age <65: 53.3 [SD: 8.0] years; age ≥65: 70.8 [5.2] years vs commercial: 45.7 [10.2] years) and lived in more rural locations (age <65 years: 43.5%, age ≥65 years: 47.7% vs commercial: 35.1%). More commercially insured patients received DMTs than Medicare Advantage patients (67.3% vs 41.5%). Among Medicare Advantage patients, 51.1% of patients younger than 65 years received DMTs, and only 27.7% of patients 65 years and older received DMTs.

### Multiple Sclerosis–Related Conditions and Symptoms

The most common conditions and symptoms were malaise and fatigue (37.3%), abnormality of gait (29.4%), burning/numbness/tingling sensations (26.4%), fibromyalgia/myalgia and myositis (12.5%), and urinary incontinence (12.2%) (**Table 2**). Malaise and fatigue, fibromyalgia/myalgia and myositis, spasms, convulsions, and voice disturbances were more common among Medicare Advantage beneficiaries younger than 65 years (all *P* < .01). Abnormality of gait, urinary continence, ataxia, fecal incontinence, and trigeminal neuralgia were more common among Medicare Advantage beneficiaries 65 years and older (all *P* < .0001). Burning, numbness, and tingling sensations were more common among commercially insured patients (*P* < .0001).

**Table 2.** Multiple Sclerosis–Related Conditions and Symptoms in Patients With MS by Payer Type^a^

**Comorbidity**	**Overall** **(N = 5000)**	**Payer Type^b^**			
		**Commercial (n = 2667)**	**Medicare Advantage**		
			**Overall** **(n = 2333)**	**Age <65 y** **(n = 1382)**	**Age ≥65 y (n = 951)**
Abnormality of gait	29.4	18.4	42.1	40.6	44.2
Ataxia	10.0	7.3	13.2	12.5	14.2
Burning, numbness, tingling sensations	26.4	31.9	20.1	22.6	16.5
Convulsions	3.1	1.8	4.5	4.9	4.0
Depression	23.2	15.7	31.7	37.0	24.1
Fecal incontinence	2.5	1.5	3.6	3.1	4.3
Fibromyalgia/myalgia and myositis	12.5	11.0	14.1	16.2	11.2
Malaise and fatigue	37.3	32.3	43.0	45.1	40.1
Optic neuritis (NS)	1.0	1.2	0.9	0.9	0.7
Spasms	9.9	7.3	12.9	15.1	9.8
Trigeminal neuralgia	2.5	1.5	3.6	3.0	4.4
Urinary incontinence	12.2	6.5	18.8	17.7	20.4
Voice disturbances	1.8	1.3	2.4	2.6	2.1

Abbreviations: MS, multiple sclerosis; NS, not significant.

^a^Unless otherwise noted, all data are reported as percentage of patients.

^b^The comparisons among commercial, Medicare Advantage patients age <65 years, and Medicare Advantage patients ≥65 years were statistically significant at *P* < .05 for all comorbidities except optic neuritis.

### Comorbidities and Multimorbidity in Patients With MS

**Figure 2.**
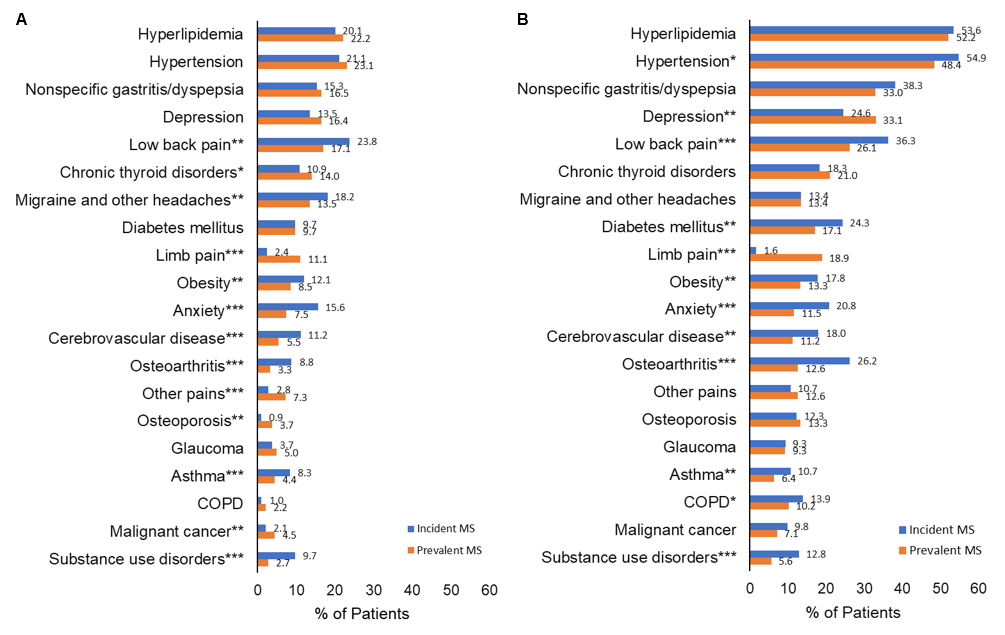
Top 20 Most Common Comorbidities by Payer Type and Incident or Prevalent MS Cases Abbreviations: COPD, chronic obstructive pulmonary disease; MS, multiple sclerosis. *P < .05. **P < .01. ***P < .0001.

**Table 3** shows the most common comorbid conditions in ranked order by payer type. The 20 most common comorbidities by newly diagnosed patients with MS vs patients with prevalent MS are presented by payer type in **Figure 2** and for the overall cohort in the **Online Supplementary Material, Figure S2**. The mean (SD) number of comorbidities in patients with incident MS was similar to that of patients with prevalent MS (4.9 [3.8] vs 4.8 [3.6], *P* = .8971). The mean (SD) CCI score for the entire cohort was 0.92 (1.49). The mean CCI score was higher in Medicare Advantage patients than in commercially insured patients (1.37 [1.79] vs 0.53 [1.02], *P* < .0001). Among Medicare Advantage patients, CCI was higher in patients 65 years and older than those younger than 65 years (1.65 [1.95] vs 1.17 [1.64], *P* < .0001). The prevalence of CCI comorbidities overall and by payer type are presented in **Online Supplementary Material, Table S2**.

**Table 3.** Most Common Comorbid Conditions in Patients With MS by Payer Type^a^

**Comorbidity**	**Overall** **(N = 5000)**	**Payer Type^b^**			
		**Commercial (n = 2667)**	**Medicare Advantage**		
			**Overall** **(n = 2333)**	**Age <65 y** **(n = 1382)**	**Age ≥65 y (n = 951)**
Hyperlipidemia	36.1	21.9	52.4	44.4	63.9
Hypertension	35.1	22.6	49.4	40.9	61.8
Nonspecific gastritis/dyspepsia	24.4	16.2	33.8	31.0	37.9
Depression	23.2	15.7	31.7	37.0	24.1
Low back pain	23.0	18.8	27.7	29.8	24.7
Chronic thyroid disorders	16.7	13.2	20.6	18.1	24.2
Migraine and other headaches	14.1	14.7	13.4	17.8	6.9
Diabetes mellitus	13.7	9.7	18.2	16.4	20.9
Limb pain	12.3	8.9	16.2	16.3	16.1
Obesity	11.5	9.4	14.0	15.3	12.0
Anxiety	11.1	9.5	13.0	13.9	11.7
Cerebrovascular disease	9.4	6.9	12.3	9.8	15.8
Osteoarthritis	9.4	4.7	14.7	11.4	19.7
Other pains	9.0	6.2	12.3	13.0	11.2
Osteoporosis	7.7	3.0	13.2	7.2	21.8
Glaucoma	6.8	4.7	9.3	6.3	13.6
Asthma	6.2	5.4	7.0	7.9	5.8
COPD	6.0	1.9	10.8	10.3	11.5
Malignant cancer	5.6	3.9	7.5	5.6	10.3
Substance use disorders	5.5	4.5	6.7	8.9	3.6
Peripheral vascular disease	5.1	1.7	8.9	5.0	14.6
Ischemic heart disease	4.6	1.3	8.3	5.5	12.3
Dementia	3.8	1.2	6.8	4.8	9.9
Metabolic syndrome	3.2	2.4	4.2	3.9	4.6
Congestive heart failure	3.2	0.8	6.0	4.2	8.6
Chronic renal failure	2.9	0.9	5.2	3.5	7.7

Abbreviations: COPD, chronic obstructive pulmonary disease; MS, multiple sclerosis.

^a^Unless otherwise noted, all data are reported as percentage of patients.

^b^The comparisons among commercial, Medicare Advantage age <65 years, and Medicare Advantage age ≥65 years were statistically significant at *P* < .05 for all comorbidities.

Vascular comorbidities were the most common comorbid condition; the most common vascular comorbidities were hyperlipidemia (36.1%), hypertension (35.1%), peripheral vascular disease (5.1%), and ischemic heart disease (4.6%). Hyperlipidemia, hypertension, peripheral vascular disease, and ischemic heart disease were all more common in Medicare Advantage patients than in commercially insured patients (52.4% vs 21.9%, 49.4% vs 22.6%, 8.9% vs 1.7%, 8.3% vs 1.3%, respectively; all *P* < .0001). Among Medicare Advantage patients, all 4 of these comorbidities were more common in patients age 65 and older than those younger than age 65 (all *P* < .0001).

The most common autoimmune comorbidities were chronic thyroid disorder (16.7%), asthma (6.2%), and IBD (1.7%). Chronic thyroid disorder was more common in Medicare Advantage patients than in commercially insured patients (20.6% vs 13.2%, *P* < .0001), and among Medicare Advantage patients, chronic thyroid disorder was more common in patients 65 years and older than in those younger than age 65 (24.2% vs 18.1%, *P* < .0001). Asthma was more common in Medicare Advantage patients than in commercially insured patients (7.0% vs 5.4%, *P* = .0168), and among Medicare Advantage patients, asthma was more common in patients younger than 65 than in patients aged 65 or older (7.9% vs 5.8%, *P* = .0408). There was no difference in IBD among the 3 groups (*P* = .0590). Of all patients, 13.7% had some form of diabetes mellitus. Diabetes mellitus was more common in Medicare Advantage patients than commercially insured patients (18.2% vs 9.7%, *P* < .0001), and among Medicare Advantage patients, diabetes mellitus was more common in patients aged 65 and older than those younger than age 65 (20.9% vs 16.4%, *P* = .0054).

The most common psychiatric comorbidities were depression (23.2%), anxiety (11.1%), and bipolar disorder (2.5%). All 3 comorbidities were more common in Medicare Advantage patients than in commercially insured patients (31.7% vs 15.7%, 13.0% vs 9.5%, 3.6% vs 1.5%, respectively; all *P* < .0001). Among Medicare Advantage patients, depression, anxiety, and bipolar disorder were more common in patients younger than 65 than in patients 65 or older (36.9% vs 24.1%, 13.9% vs 11.7%, 5.0% vs 1.7%, respectively; *P* < .0001, *P* = .0147, *P* < .0001, respectively).

**Figure 3.**
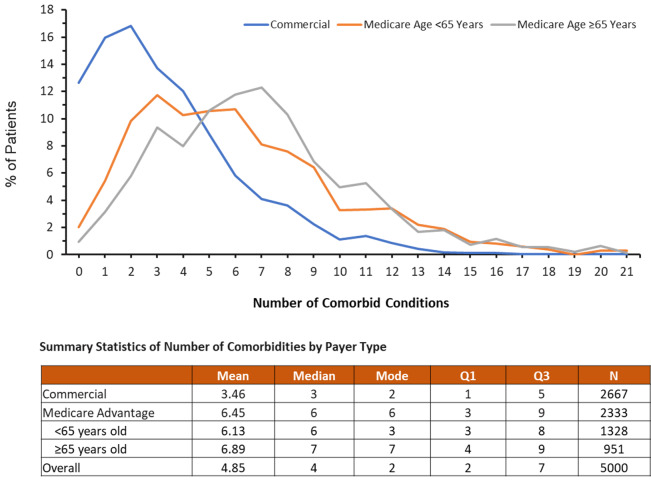
Distribution of Number of Comorbid Conditions in Patients With MS by Payer Type Abbreviations: MS, multiple sclerosis; Q1, 25th percentile of the data; Q3, 75th percentile of the data.

**Figure 3** shows the distribution of the number of comorbid conditions in patients with MS by payer type. Across the cohort, 75.7% (71.5% of commercially insured patients, 93.9% of Medicare Advantage patients) had 2 or more comorbidities, 63.8% had 3 or more comorbidities, and 42.2% had 5 or more comorbidities (**Online Supplementary Material, Figure S3**). The mean (SD) number of comorbidities among patients with MS was 4.9 (3.7). There were more comorbidities in Medicare Advantage patients than in commercially insured patients (6.5 [3.8] vs 3.5 [2.9], *P* < .0001). Among Medicare Advantage patients, there was no difference in number of comorbidities between patients younger than 65 years and those 65 years and older (6.9 [3.7] vs 6.1 [3.9], *P* = .2009).

## DISCUSSION

Data regarding the wide spectrum of comorbidities among patients with MS are still scarce and only 2 published studies were large US population-based evaluations.^10,11^ In the present study, patient characteristics, comorbidities, and multimorbidity in both Medicare Advantage and commercially insured populations were evaluated using a large administrative healthcare claims database. Patient characteristics observed in this study are consistent with the published literature.^13,18,30,31^

Multiple sclerosis–specific conditions and symptoms (ie, malaise, fatigue, depression/anxiety, spasms, fibromyalgia, convulsions) were more common among Medicare Advantage beneficiaries younger than age 65 (all *P* < .0001). Our study demonstrated that hyperlipidemia and hypertension were among the most common comorbidities in MS, presenting in 36.1% and 35.1% of patients, respectively; the most prevalent comorbid autoimmune disease was chronic thyroid disorder (16.7%); the most common gastrointestinal disease was nonspecific gastritis/dyspepsia (24.4%); and the most common psychiatric comorbidities were depression (23.2%), anxiety (11.1%), and bipolar disorder (2.5%).

Some of our estimates were higher than recent meta-analysis estimates (hyperlipidemia, 10.9%; hypertension, 18.6%; thyroid disorder, 6.4%), the prevalence of depression was similar (meta-analysis estimate, 23.7%), and some were lower than the meta-analysis estimates (anxiety, 21.9%; bipolar disorder, 5.8%).^8^ These discrepancies may be due to differences in the patient populations evaluated, the study designs, data sources, and the methods used for evaluating comorbidities. Hence, these findings highlight that it is difficult to make comparisons across studies. A US commercial claims analysis by Edwards et al^11^ reported that common comorbidities among patients with MS were hyperlipidemia and hypertension (range, 25.9%-29.7% per year from 2006 to 2014), gastrointestinal disease (range, 18.4%-21.2%), and thyroid disease (range, 12.9%-17.1%). Chronic lung disease, arthritis, anxiety, diabetes, and depression were present in approximately 5% to 10% of US patients with MS.^11^ A previous study of Medicare patients by Gilden et al^32^ evaluated data from 2003 to 2006 and found that the rates of the most common comorbid conditions for prevalent progressive MS and prevalent relapsing remitting MS (RRMS) cases, respectively, were depression (51.7%, 24.4%), ischemic heart disease (31.1%, 12.1%), diabetes (28.6%, 13.3%), chronic obstructive pulmonary disease (28.0%, 12.9%), cardiovascular disease (24.3%, 6.5%), and arthritis (22.5%, 15.5%). These rates are relatively consistent with the findings of the current study when considering the weighting of patients with progressive MS vs RRMS. The rates of hypertension and hyperlipidemia in the current study are higher than the rate of cardiovascular disease reported by Gilden et al.^32^ This may be due to their data being approximately 15 years old and cardiovascular disease being more prevalent and more commonly diagnosed in more recent years.

Vascular comorbidities such as hyperlipidemia and hypertension are particularly noteworthy as they have been shown to be associated with increased risk of disability progression in MS.^8^ A cohort study using the North American Research Committee on Multiple Sclerosis (NARCOMS) registry found that on average, patients with MS with vascular comorbidities progressed to a score of 6 on the Expanded Disability Status Scale (EDSS) 6 years faster than patients with MS without a vascular comorbidity^2^ and that these comorbidities affected visual disability.^33^ A Swedish cohort study found that patients with MS with depression showed a significantly higher risk of progression on the EDSS score.^34^ Among NARCOMS registry participants, vascular, mental, and visual comorbidities increased the risk of mortality.^35^ Depression and MS have synergistic effects on mortality, highlighting the importance of preventing and treating depression.^5,36^

Multiple comorbidities, which are highly prevalent among patients with MS, adversely affected a broad range of outcomes.^9,37,38^ Our study demonstrated that 75.7% of patients with MS had at least 2 comorbidities, 63.4% had 3 or more comorbidities, and 42.2% had 5 or more comorbidities. There were more comorbidities in Medicare Advantage patients than in commercially insured patients (6.5 vs 3.5); however, among Medicare Advantage patients with MS, there was no difference in the number of comorbidities between younger-than-65 years and 65-years-and-older groups. In a Canadian survey data analysis, Warren et al^39^ found that the mean number of comorbidities in patients with MS was 1.6 and that 10% of patients had 8 or more comorbidities. The study design could be the reason for the different findings between our study and the survey data analysis. The administrative claims data include more comprehensive information, especially comorbidities, than the health survey data, due to the limited space of the survey questionnaires or instruments, which usually contain highly selected comorbid conditions.

Comorbidities increase the complexity of disease management and pose major challenges.^16,23,29^ Several studies have found that comorbidities delay MS diagnosis from symptom onset.^1,37^ A prospective multicenter cohort study conducted in 4 Canadian MS clinics reported that, compared with those without any comorbidity, relapse rate increased over the next 2 years in patients with 3 or more comorbidities at baseline.^3^ Another prospective cohort study found that fatigue and the presence of 3 or more physical comorbidities were significantly associated with higher rates of physician visits, prescriptions filled, and hospitalizations in patients with MS.^40^ A large Canadian study of 10 698 patients found that the likelihood of initiating DMT decreased with an increasing number of comorbidities.^41^ This is consistent with our findings that only 41.5% of all Medicare Advantage patients and only 27.7% of Medicare Advantage patients 65 years and older were receiving a DMT, compared with 67.3% of commercially insured patients. In the above-mentioned Canadian study, comorbid anxiety and ischemic heart disease were shown to be associated with reduced initiation of DMT. However, patients with depression were 13% more likely to initiate DMT compared with those without depression at the index date.^41^ Comorbidities have also been shown to be associated with a higher risk of switching from the first DMT due to intolerance.^42^

Our data show a wide spectrum of comorbidities among patients with MS, supporting that MS disease management should be individualized and tailored to specific needs for each patient. Patients with MS with multiple comorbidities may have many healthcare providers; without proper coordination, this could lead to fragmented care, delay or duplication of healthcare services, inappropriate medications, polypharmacy, and adverse drug interactions.^16,23^ Literature regarding the impact of comorbidity and multimorbidity on MS treatment is still limited. Further study should focus on the impact of comorbidity and multimorbidity on MS treatment/intervention and outcomes in the various subgroups of patients.

### Limitations

Several limitations may affect interpretation of these results. First, this administrative claims data analysis may have underestimated the prevalence rates of some comorbidities and multimorbidity.^16,23^ Second, only descriptive statistics were provided, and comparisons between groups should be interpreted with caution. No adjusted analyses were conducted to control for confounding factors. Third, there are no standard operational definitions for comorbidity,^23,43,44^ so we included the 59 most common comorbidities and excluded rarer conditions. Since classification of all comorbidities was based on ICD-10-CM codes alone, misclassification is likely. However, this approach is commonly used in other studies of comorbidities.^5,11,16,23–26,44^ Fourth, there are no ICD-10-CM codes specific to MS subtypes, so we cannot differentiate RRMS from secondary progressive MS. Fifth, the study results may not be generalizable to patients with other health insurance or healthcare, such as those with traditional Medicare, the uninsured, and patients in other countries.

## CONCLUSIONS

Multiple sclerosis–specific conditions and symptoms (ie, malaise, fatigue, depression/anxiety, spasms, fibromyalgia, convulsions) were more common among Medicare Advantage beneficiaries aged 65 years and older. Our study demonstrated that hyperlipidemia and hypertension were among the most common comorbidities in MS. Comorbidities and multimorbidity patterns differed between patients with MS with commercial insurance and patients with the Medicare Advantage health plan. Multimorbidity is highly prevalent among patients with MS and should be considered in the context of clinical decision making to optimize MS management and improve patient outcomes.

### Author Contributions

D.D. was responsible for concept and study design, acquisition and analysis of the data, interpretation of the results, and drafting of the manuscript. A.S. was responsible for clinical concept and study design, interpretation of the results, and validation. A.L.P. was responsible for concept and study design, obtained funding, interpretation of the results, and validation. C.L. was responsible for concept and study design, obtained funding, analysis of the data, interpretation of the results, and drafting of the manuscript. All authors fully contributed to the content of this manuscript. All authors had full access to all the data in the study and take full responsibility for the integrity of the work and the accuracy of the data analysis, from inception to published article.

### Role of the Funder/Sponsor

The funders were involved in the design and conduct of the study; analysis and interpretation of the data; preparation, review, or approval of the manuscript; and decision to submit the manuscript for publication.

### Disclosures

D.D. and A.S. were employees of CVS Health at the time the study was conducted. This study was funded by EMD Serono. A.L.P. and C.L. were employees of EMD Serono at the time the study was conducted. No other disclosures were reported.

### Meeting Presentation

Initial findings from this study were presented at AMCP Nexus 2020 Virtual Conference, October 20-23, 2020. The abstract was published in: *J Manag Care Spec Pharm.* 2020;26(10-a):S44-45.

## Supplementary Material

Online Supplementary Material

## References

[ref-160396] Marrie R. A., Horwitz R., Cutter G., Tyry T., Campagnolo D., Vollmer T. (2009). Comorbidity delays diagnosis and increases disability at diagnosis in MS. Neurology.

[ref-160397] Marrie R. A., Rudick R., Horwitz R., Cutter G., Tyry T., Campagnolo D., Vollmer T. (2010). Vascular comorbidity is associated with more rapid disability progression in multiple sclerosis. Neurology.

[ref-160398] Kowalec Kaarina, McKay Kyla A., Patten Scott B., Fisk John D., Evans Charity, Tremlett Helen, Marrie Ruth Ann, For the CIHR Team in Epidemiology and Impact of Comorbidity on Multiple Sclerosis (ECoMS) (2017). Comorbidity increases the risk of relapse in multiple sclerosis: a prospective study. Neurology.

[ref-160399] Marrie R. A., Elliott L., Marriott J., Cossoy M., Tennakoon A., Yu N. (2015). Comorbidity increases the risk of hospitalizations in multiple sclerosis. Neurology.

[ref-160400] Marrie Ruth Ann, Elliott Lawrence, Marriott James, Cossoy Michael, Blanchard James, Leung Stella, Yu Nancy (2015). Effect of comorbidity on mortality in multiple sclerosis. Neurology.

[ref-160401] Wolff Jennifer L., Starfield Barbara, Anderson Gerard (2002). Prevalence, expenditures, and complications of multiple chronic conditions in the elderly. Archives of Internal Medicine.

[ref-160402] Berrigan Lindsay I., Fisk John D., Patten Scott B., Tremlett Helen, Wolfson Christina, Warren Sharon, Fiest Kirsten M., McKay Kyla A., Marrie Ruth Ann, For the CIHR Team in the Epidemiology and Impact of Comorbidity on Multiple Sclerosis (ECoMS) (2016). Health-related quality of life in multiple sclerosis: direct and indirect effects of comorbidity. Neurology.

[ref-160403] Marrie Ruth Ann, Cohen Jeffrey, Stuve Olaf, Trojano Maria, Sørensen Per Soelberg, Reingold Stephen, Cutter Gary, Reider Nadia (2015). A systematic review of the incidence and prevalence of comorbidity in multiple sclerosis: overview. Multiple Sclerosis Journal.

[ref-160404] Magyari Melinda, Sorensen Per Soelberg (2020). Comorbidity in multiple sclerosis. Frontiers in Neurology.

[ref-160405] Capkun Gorana, Dahlke Frank, Lahoz Raquel, Nordstrom Beth, Tilson Hugh H, Cutter Gary, Bischof Dorina, Moore Alan, Simeone Jason, Fraeman Kathy, Bancken Fabrice, Geissbühler Yvonne, Wagner Michael, Cohan Stanley (2015). Mortality and comorbidities in patients with multiple sclerosis compared with a population without multiple sclerosis: an observational study using the US Department of Defense administrative claims database. Multiple Sclerosis and Related Disorders.

[ref-160406] Edwards Natalie C, Munsell Michael, Menzin Joseph, Phillips Amy L (2018). Comorbidity in US patients with multiple sclerosis. Patient Related Outcome Measures.

[ref-160407] Bhattacharjee S, Yegezu Z, Kollecas K. (2020). Comorbidity pattern among adults with MS in the United States: a propensity score–matched study. Neurology.

[ref-160408] Li Pengxiang, Ladage Vrushabh P., Berger Joseph, Chahin Salim, Jhaveri Mehul, Geremakis Caroline, Doshi Jalpa A. (2020). Disease-modifying therapy adherence and associated factors in a national sample of Medicare patients with multiple sclerosis. Value in Health.

[ref-160409] Meyers David J, Johnston Kenton J. (2021). The growing importance of Medicare advantage in health policy and health services research. JAMA Health Forum.

[ref-160410] Centers for Medicare and Medicaid Services (CMS) MA State/County Penetration 2021.

[ref-160411] Dai Dingwei, Samiian Ali, Fernandes Joaquim, Coetzer Henriette (2022). Multiple comorbidities, psychiatric disorders, healthcare resource utilization and costs among patients with essential tremor: a retrospective observational study in a large US commercially insured and Medicare Advantage population. Journal of Health Economics and Outcomes Research.

[ref-160412] Wallin Mitchell T., Culpepper William J., Campbell Jonathan D., Nelson Lorene M., Langer-Gould Annette, Marrie Ruth Ann, Cutter Gary R., Kaye Wendy E., Wagner Laurie, Tremlett Helen, Buka Stephen L., Dilokthornsakul Piyameth, Topol Barbara, Chen Lie H., LaRocca Nicholas G. (2019). The prevalence of MS in the United States: a population-based estimate using health claims data. Neurology.

[ref-160413] Prescott Jeff D., Factor Saul, Pill Michael, Levi Gary W. (2007). Descriptive analysis of the direct medical costs of multiple sclerosis in 2004 using administrative claims in a large nationwide database. Journal of Managed Care Pharmacy.

[ref-160414] Marrie Ruth Ann, Miller Aaron, Sormani Maria Pia, Thompson Alan, Waubant Emmanuelle, Trojano Maria, O'Connor Paul, Fiest Kirsten, Reider Nadia, Reingold Stephen, Cohen Jeffrey A., For the attendees of the International Workshop on Comorbidity in Multiple Sclerosis (2016). Recommendations for observational studies of comorbidity in multiple sclerosis. Neurology.

[ref-160415] Valderas J. M., Starfield B., Sibbald B., Salisbury C., Roland M. (2009). Defining comorbidity: implications for understanding health and health services. The Annals of Family Medicine.

[ref-160416] van den Akker Marjan, Buntinx Frank, Knottnerus J André (1996). Comorbidity or multimorbidity. European Journal of General Practice.

[ref-160417] Marck Claudia Helena, Neate Sandra Leanne, Taylor Keryn Louise, Weiland Tracey Joy, Jelinek George Alexander (2016). Prevalence of comorbidities, overweight and obesity in an international sample of people with multiple sclerosis and associations with modifiable lifestyle factors. PLoS One.

[ref-160418] Dai Dingwei, Sharma Ajay, Alvarez Paula, Woods Steven (2022). Multiple comorbid conditions and healthcare resource utilization among adult patients with hyperkalemia: a retrospective observational cohort study using association rule mining. Journal of Multimorbidity and Comorbidity.

[ref-160419] Quan Hude, Sundararajan Vijaya, Halfon Patricia, Fong Andrew, Burnand Bernard, Luthi Jean-Christophe, Saunders L Duncan, Beck Cynthia A., Feasby Thomas E., Ghali William A. (2005). Coding algorithms for defining comorbidities in ICD-9-CM and ICD-10 administrative data. Medical Care.

[ref-160420] Charlson Mary E., Pompei Peter, Ales Kathy L., MacKenzie C.Ronald (1987). A new method of classifying prognostic comorbidity in longitudinal studies: development and validation. Journal of Chronic Diseases.

[ref-160421] Charlson Mary E., Charlson Robert E., Peterson Janey C., Marinopoulos Spyridon S., Briggs William M., Hollenberg James P. (2008). The Charlson comorbidity index is adapted to predict costs of chronic disease in primary care patients. Journal of Clinical Epidemiology.

[ref-160422] Sundararajan V., Henderson T., Perry C., Muggivan A., Quan H., Ghali W.A. (2004). New ICD-10 version of the Charlson comorbidity index predicted in-hospital mortality. J Clin Epidemiol.

[ref-160423] Boyd Cynthia M., Fortin Martin (2010). Future of multimorbidity research: how should understanding of multimorbidity inform health system design?. Public Health Reviews.

[ref-160424] Academy of Medical Sciences (2018). Multimorbidity: A priority for global health research.

[ref-160425] Dilokthornsakul Piyameth, Valuck Robert J., Nair Kavita V., Corboy John R., Allen Richard R., Campbell Jonathan D. (2016). Multiple sclerosis prevalence in the United States commercially insured population. Neurology.

[ref-160426] Nazareth Tara, Datar Manasi, Yu Tzy-Chyi (2019). Treatment effectiveness for resolution of multiple sclerosis relapse in a US health plan population. Neurology and Therapy.

[ref-160427] Gilden Daniel M., Kubisiak Joanna, Zbrozek Arthur S. (2011). The economic burden of Medicare-eligible patients by multiple sclerosis type. Value in Health.

[ref-160428] Marrie Ruth Ann, Cutter Gary, Tyry Tuula (2011). Substantial adverse association of visual and vascular comorbidities on visual disability in multiple sclerosis. Multiple Sclerosis Journal.

[ref-160429] Binzer Stefanie, McKay Kyla A., Brenner Philip, Hillert Jan, Manouchehrinia Ali (2019). Disability worsening among persons with multiple sclerosis and depression: a Swedish cohort study. Neurology.

[ref-160430] Salter Amber, Tyry Tuula, Wang Guoqiao, Fox Robert J., Cutter Gary, Marrie Ruth Ann (2016). Examining the joint effect of disability, health behaviors, and comorbidity on mortality in MS. Neurology: Clinical Practice.

[ref-160431] Marrie Ruth Ann, Walld Randy, Bolton James M., Sareen Jitender, Patten Scott B., Singer Alexander, Lix Lisa M., Hitchon Carol A., El-Gabalawy Renée, Katz Alan, Fisk John D., Bernstein Charles N. (2018). Psychiatric comorbidity increases mortality in immune-mediated inflammatory diseases. General Hospital Psychiatry.

[ref-160432] Marrie Ruth Ann (2017). Comorbidity in multiple sclerosis: implications for patient care. Nature Reviews Neurology.

[ref-160433] Marrie Ruth Ann (2019). Comorbidity in multiple sclerosis: past, present and future. Clinical and Investigative Medicine.

[ref-160434] Warren Sharon A., Turpin Karen V.L., Pohar Sheri L., Jones C. Allyson, Warren K.G. (2009). Comorbidity and health-related quality of life in people with multiple sclerosis. International Journal of MS Care.

[ref-160435] McKay Kyla A., Marrie Ruth Ann, Fisk John D., Patten Scott B., Tremlett Helen (2018). Comorbidities are associated with altered health services use in multiple sclerosis: a prospective cohort study. Neuroepidemiology.

[ref-160436] Zhang Tingting, Tremlett Helen, Leung Stella, Zhu Feng, Kingwell Elaine, Fisk John D., Bhan Virender, Campbell Trudy L., Stadnyk Karen, Yu B. Nancy, Marrie Ruth Ann, For the CIHR Team in the Epidemiology and Impact of Comorbidity on Multiple Sclerosis (2016). Examining the effects of comorbidities on disease-modifying therapy use in multiple sclerosis. Neurology.

[ref-160437] Laroni Alice, Signori Alessio, Maniscalco Giorgia T., Lanzillo Roberta, Russo Cinzia Valeria, Binello Eleonora, Lo Fermo Salvatore, Repice Annamaria, Annovazzi Pietro, Bonavita Simona, Clerico Marinella, Baroncini Damiano, Prosperini Luca, La Gioia Sara, Rossi Silvia, Cocco Eleonora, Frau Jessica, Torri Clerici Valentina, Signoriello Elisabetta, Sartori Arianna, Zarbo Ignazio Roberto, Rasia Sarah, Cordioli Cinzia, Cerqua Raffaella, Di Sapio Alessia, Lavorgna Luigi, Pontecorvo Simona, Barrilà Caterina, Saccà Francesco, Frigeni Barbara, Esposito Sabrina, Ippolito Domenico, Gallo Fabio, Sormani Maria Pia (2017). Assessing association of comorbidities with treatment choice and persistence in MS: a real-life multicenter study. Neurology.

[ref-160438] Diederichs C., Berger K., Bartels D. B. (2011). The measurement of multiple chronic diseases—a systematic review on existing multimorbidity indices. The Journals of Gerontology Series A: Biological Sciences and Medical Sciences.

[ref-160439] Kern David M., Cepeda M. Soledad (2020). Treatment patterns and comorbid burden of patients newly diagnosed with multiple sclerosis in the United States. BMC Neurology.

