# Supplementary material for: Patterns of Comorbidity and Multimorbidity Among Patients With Multiple Sclerosis in a Large US Commercially Insured and Medicare Advantage Population
Source: J Health Econ Outcomes Res. 2022 Nov 21;9(2):125–33. doi: 10.36469/001c.38669 (PMC9684016; doi:10.36469/001c.38669)
Supplement: Online Supplementary Material [file jheor_2022_9_2_38669_120816.pdf]

Journal of Health Economics and Outcomes Research

Online Supplementary Material

Patterns of Comorbidity and Multimorbidity Among Patients With Multiple Sclerosis in a Large US Commercially Insured and Medicare Advantage Population. *JHEOR*. 2022;9(2):125-133. doi:10.36469/jheor.2022.38669

Table S1: List of ICD-10-CM Codes for Comorbid Conditions

Table S2: Charlson Comorbid Conditions in Patients with MS by Payer Type

Figure S1: Study Design Diagram

Figure S2: Top 20 Most Common Comorbidities by Incident or Prevalent Multiple Sclerosis

Figure S3: Summed Proportions of Multimorbidity With the Indicated Number of Comorbid Conditions Among Patients With Multiple Sclerosis by Payer Type

This supplementary material has been provided by the authors to give readers additional information about their work.

This is an open-access article distributed under the terms of the Creative Commons Attribution 4.0 International License (CCBY-4.0). View this license's legal deed at http://creativecommons.org/licenses/by/4.0 and legal code at http://creativecommons.org/licenses/by/4.0/legalcode for more information.

Table S1. List of ICD-10-CM Codes for Comorbid Conditions

Comorbid Conditions	ICD-10-CM Code(s)
Abnormality of gait	R26.x
Ataxia	R27.0, R27.8, R27.9
Atrial fibrillation	I48.0–I48.2, I48.4, I48.91
AIDS/HIV	B20.x–B22.x, B24.x
Anxiety	F40.x F41.x
Asthma	J45.2–J45.5, J45.90
Bipolar disorder	F31.x
Cancer (malignant)	C00.x–C97.x
Cerebrovascular disease	G45.x, G46.x, I60.x–I69.x, H34.0
Cholelithiasis/cholecystitis	K80.x
Chronic obstructive pulmonary disease	J41.x–J44.x
Chronic renal failure	N18.5, N18.6, N19.x
Chronic thyroid disorders	E00.x–E03.x, E06.5, E07.9, E89.0
Congenital heart disease	Q20.x–Q28.x
Congestive heart failure	I09.9, I11.0, I13.0, I13.2, I25.5, I42.0, I42.5–I42.9, I43.x, I50.x, P29.0
Connective tissue disorder	M31.5, M32.x - M34.x, M35.1, M35.3, M36.0
Convulsions	F44.5, R56.9
Dementia	F00.x–F03.x, F05.1, G30.x, G31.1
Depression	F20.4, F31.3–F31.5, F32.x, F33.x, F34.1, F41.2, F43.2
Diabetes mellitus	E10.x–E14.x
Diverticular disease	K57.x
Epilepsy	G40.x
Fecal incontinence	R15.x
Fibromyalgia	M79.7x
Glaucoma	H40.x, H42.x
Hypercoagulable syndrome	D68.59, D68.69
Chronic hepatitis	K73.x, K75.x
Hyperlipidemia	E78.0-E78.5
Hypertension	I10.x–I13.x, I15.x
Inflammatory bowel disease	K50.x–K52.x
Iron deficiency anemia	D50.8, D50.9
Ischemic heart disease	I20.x–I25.x
Kidney stones	N20.x
Low back pain	M54.5
Limb pain	M79.6x
Malaise and fatigue	R35.x
Metabolic syndrome	E88.81
Migraine and other headaches	G43.x, G44.x
Nonspecific gastritis/dyspepsia	K29.70, K30.x
Obesity	E66.x
Optic neuritis	H46.x, G36.0
Osteoarthritis	M15.x–M19.x
Osteoporosis	M80.x, M81.x

Table S1. List of ICD-10-CM Codes for Comorbid Conditions, *cont'd*

Comorbid Conditions	ICD-10-CM Code(s)
Other pains	E08.40, E08.42, E09.40, E09.42, E10.40, E10.42, E11.40, E11.42, E13.40, E13.42, M514.12, M514.16, M514.17, R10
Pancreatitis	K85.x
Paraplegia and hemiplegia	G04.1, G11.4, G80.1, G80.2, G81.x, G82.x, G83.0 - G83.4, G83.9
Parkinson's disease	G20.x
Psychoses	F20.x, F22.x–F25.x, F28.x, F29.x, F30.2, F31.2, F31.5
Peptic ulcer disease	K25.x- K28.x
Peripheral vascular disease	I70.x, I71.x, I71.1, I73.1, I73.8, I73.9, I77.1, I79.0, I79.2, K55.1, K55.8, K55.9, Z95.8, Z95.9
Rheumatoid arthritis	M05.x, M06.x
Urinary incontinence	N39.3, N39.4xR39.81
Sickle cell anemia	D57.x
Spasms	M62.83x
Substance use disorders	F10.129, F10.229, F10.929, F10.10, F10.20, F11.10, F11.20, F11.129, F11.229, F11.929, F11.122, F11.222, F11.922, F11.23, F11.99, F12.10, F12.20, F13.10, F13.20, F14.10, F14.20, F15.10, F15.20, F15.929, F16.10, F16.20, F17.200, F18.10, F18.20, F19.10, F19.20, Z72.0
Systemic lupus erythematosus	M32.x
Trigeminal neuralgia	G50.0
Voice disturbances	R47.81, R47.82, R47.89, R49.8, R49.9
Ventricular arrhythmia	I47.x

Abbreviations: AIDS, acquired immunodeficiency syndrome; HIV, human immunodeficiency virus; ICD-10-CM, *International Classification of Diseases, Tenth Revision, Clinical Modification*.

Table S2. Charlson Comorbid Conditions in Patients With MS by Payer Type[a]

Comorbidity	Overall (N = 5000)	Payer Type[b]			
		Commercial (n = 2667)	Medicare Advantage		
			Overall (n = 2333)	Age <65 y (n = 1382)	Age ≥65 y (n = 951)
Myocardial infarction	1.9	0.6	3.5	2.8	4.6
Congestive heart failure	3.2	0.8	6.0	4.2	8.6
Peripheral vascular disease	5.1	1.7	8.9	5.0	14.6
Cerebral vascular disease	9.4	6.9	12.3	9.8	15.9
Dementia	3.8	1.2	6.8	4.8	9.9
Chronic pulmonary disease	13.7	8.3	19.9	20.8	18.6
Connective tissue disorder	2.6	2.1	3.3	3.3	3.3
Peptic ulcer	1.1	0.5	1.8	1.7	2.1
Mild liver disease	2.0	1.5	2.5	2.6	2.4
Diabetes without complications	13.3	9.4	17.9	16.1	20.4
Diabetes with complications	4.0	2.0	6.3	5.5	7.6
Paraplegia and hemiplegia	7.1	4.1	10.5	10.7	10.3
Renal disease	3.9	1.5	6.6	4.1	10.3
Cancer	5.4	3.8	7.3	5.3	10.2
Metastatic cancer	0.6	0.3	0.9	0.6	1.4
Severe liver disease	0.2	0.0	0.3	0.5	0.1
HIV (NS)	0.1	0.2	0.1	0.1	0 .0

Abbreviations: HIV, human immunodeficiency virus; MS, multiple sclerosis; NS, not significant.
[a]Unless otherwise noted, all data are reported as percentage of patients.
[b]The comparisons among commercial, Medicare Advantage age <65, and Medicare Advantage age ≥65 were statistically significant at $P<.05$ except for HIV.

Figure S1. Study Design Diagram

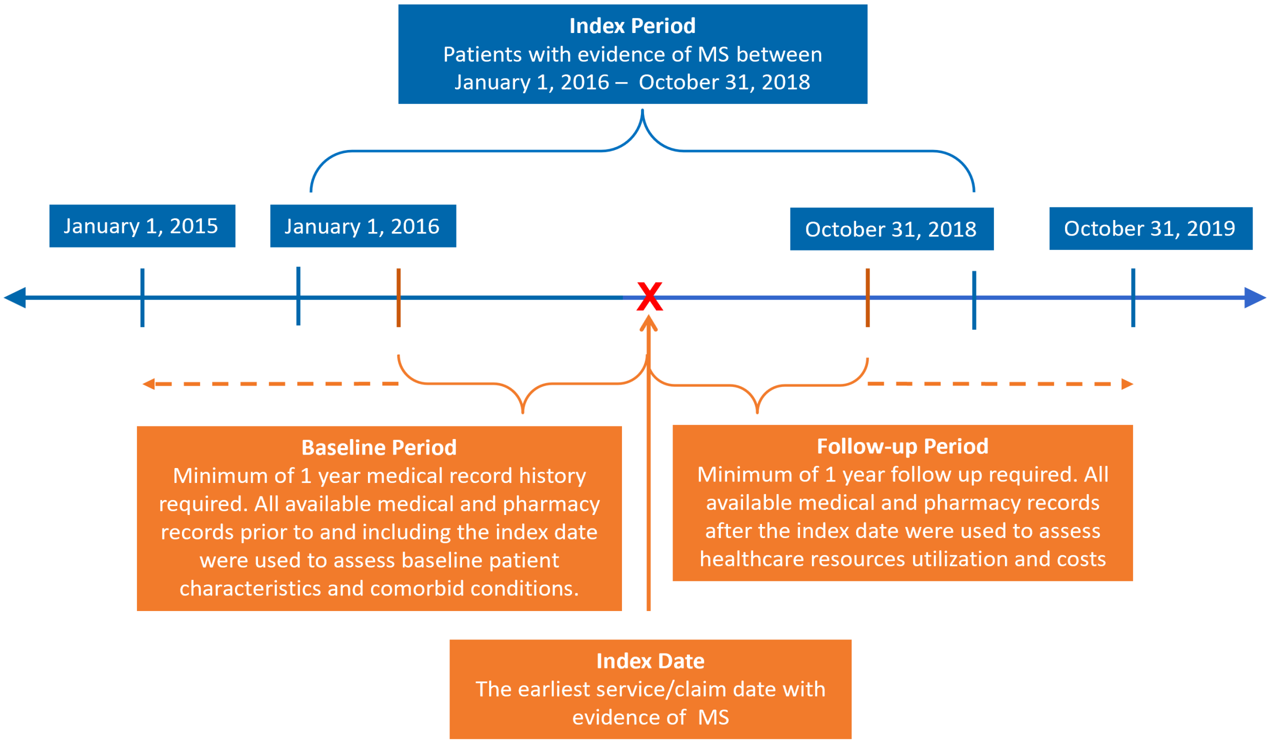

Abbreviation: MS, multiple sclerosis.

Figure S2. Twenty Most Common Comorbidities by Incident or Prevalent Multiple Sclerosis

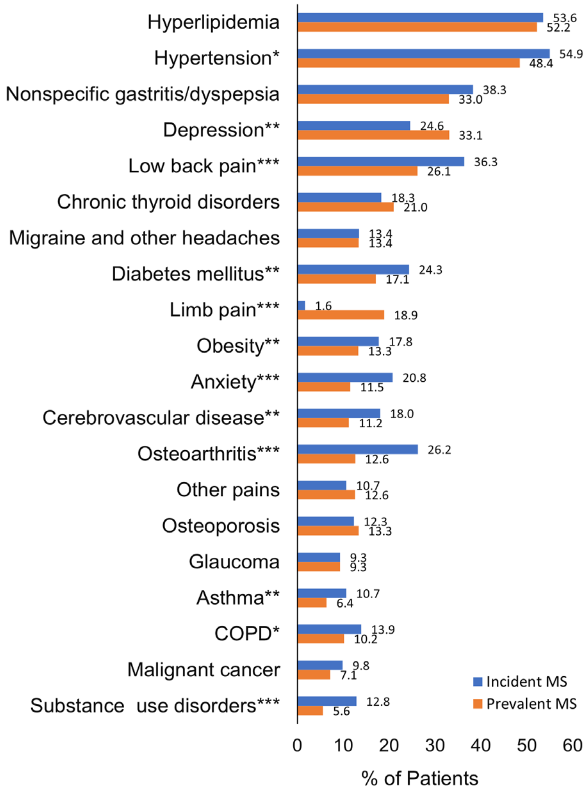

Abbreviations: COPD, chronic obstructive pulmonary disease; MS, multiple sclerosis.
$^*P<.05$, $^{**}P<.01$, $^{***}P<.0001$.

Figure S3. Summed Proportions of Multimorbidity With the Indicated Number of Comorbid Conditions Among Patients With Multiple Sclerosis by Payer Type

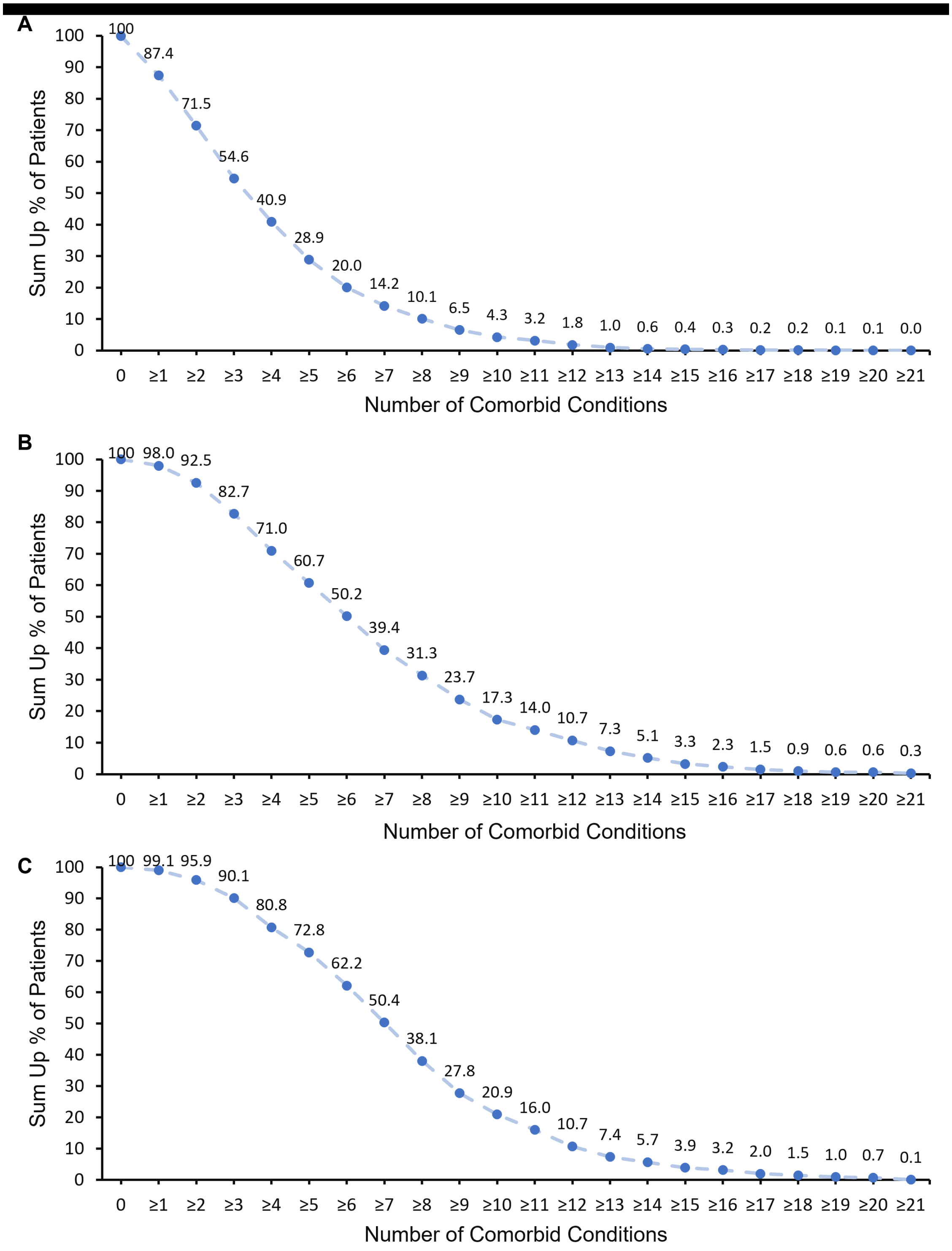

A: Commercial insurance; **B**: Medicare Advantage age <65; **C**: Medicare Advantage age ≥65.
